# Supplementary material for: COZOID: contact zone identifier for visual analysis of protein-protein interactions
Source: BMC Bioinformatics. 2018 Apr 6;19:125. doi: 10.1186/s12859-018-2113-6 (PMC5889581; doi:10.1186/s12859-018-2113-6)
Supplement: Supplementary file 2 — Software build. Executable binary file of the software tool. (ZIP 118,784 kb) [file 12859_2018_2113_MOESM2_ESM.zip › cozoid/java/docs/beansbinding-1.2.1-doc.zip!/org/jdesktop/beansbinding/Bindings.html]

Bindings (Beans Binding)


---


|  |  |  |
| --- | --- | --- |
| **Overview** \| **Package** \| **Class** \| **Use** \| **Tree** \| **Deprecated** \| **Index** \| **Help** | |  |
| **PREV CLASS**   **NEXT CLASS** | **FRAMES**    **NO FRAMES**     **All Classes** |  |
| SUMMARY: NESTED \| FIELD \| CONSTR \| METHOD | DETAIL: FIELD \| CONSTR \| METHOD |  |


---


## org.jdesktop.beansbinding Class Bindings

```
java.lang.Object
  org.jdesktop.beansbinding.Bindings
```

---

``` public class Bindings extends java.lang.Object ```

A factory class for creating instances of the concrete `Binding`
implementations provided by this package.

---

| **Method Summary** | |
| --- | --- |
| `static <SS,SV,TS,TV> AutoBinding<SS,SV,TS,TV>` | `createAutoBinding(AutoBinding.UpdateStrategy strategy, SS sourceObject, Property<SS,SV> sourceProperty, TS targetObject, Property<TS,TV> targetProperty)`             Creates an instance of `AutoBinding` that binds a property of a source object to a property of a target object. |
| `static <SS,SV,TS,TV> AutoBinding<SS,SV,TS,TV>` | `createAutoBinding(AutoBinding.UpdateStrategy strategy, SS sourceObject, Property<SS,SV> sourceProperty, TS targetObject, Property<TS,TV> targetProperty, java.lang.String name)`             Creates a named instance of `AutoBinding` that binds a property of a source object to a property of a target object. |
| `static <SS,TS,TV> AutoBinding<SS,SS,TS,TV>` | `createAutoBinding(AutoBinding.UpdateStrategy strategy, SS sourceObject, TS targetObject, Property<TS,TV> targetProperty)`             Creates an instance of `AutoBinding` that binds a source object to a property of a target object. |
| `static <SS,TS,TV> AutoBinding<SS,SS,TS,TV>` | `createAutoBinding(AutoBinding.UpdateStrategy strategy, SS sourceObject, TS targetObject, Property<TS,TV> targetProperty, java.lang.String name)`             Creates a named instance of `AutoBinding` that binds a source object to a property of a target object. |

**Methods inherited from class java.lang.Object**

`clone, equals, finalize, getClass, hashCode, notify, notifyAll, toString, wait, wait, wait`

**Method Detail**

### createAutoBinding

```
public static <SS,TS,TV> AutoBinding<SS,SS,TS,TV> createAutoBinding(AutoBinding.UpdateStrategy strategy,
                                                                    SS sourceObject,
                                                                    TS targetObject,
                                                                    Property<TS,TV> targetProperty)
```

:   Creates an instance of `AutoBinding` that binds a source object to a property of a target object.
    The `AutoBinding's` source property is set to an instance of `ObjectProperty` so that the
    source object is used directly, rather than some property of the source object.

    :   **Parameters:**: `strategy` - the update strategy for the binding: `sourceObject` - the source object: `targetObject` - the target object: `targetProperty` - the target property **Returns:**: an `AutoBinding` that binds the source object to the target property of the target object **Throws:**: `java.lang.IllegalArgumentException` - if the update strategy or target property is `null`

---


### createAutoBinding

```
public static <SS,TS,TV> AutoBinding<SS,SS,TS,TV> createAutoBinding(AutoBinding.UpdateStrategy strategy,
                                                                    SS sourceObject,
                                                                    TS targetObject,
                                                                    Property<TS,TV> targetProperty,
                                                                    java.lang.String name)
```

:   Creates a named instance of `AutoBinding` that binds a source object to a property of a target object.
    The `AutoBinding's` source property is set to an instance of `ObjectProperty` so that the
    source object is used directly, rather than some property of the source object.

    :   **Parameters:**: `strategy` - the update strategy for the binding: `sourceObject` - the source object: `targetObject` - the target object: `targetProperty` - the target property: `name` - a name for the binding **Returns:**: an `AutoBinding` that binds the source object to the target property of the target object **Throws:**: `java.lang.IllegalArgumentException` - if the update strategy or target property is `null`

---


### createAutoBinding

```
public static <SS,SV,TS,TV> AutoBinding<SS,SV,TS,TV> createAutoBinding(AutoBinding.UpdateStrategy strategy,
                                                                       SS sourceObject,
                                                                       Property<SS,SV> sourceProperty,
                                                                       TS targetObject,
                                                                       Property<TS,TV> targetProperty)
```

:   Creates an instance of `AutoBinding` that binds a property of a source object to a property of a target object.

    :   **Parameters:**: `strategy` - the update strategy for the binding: `sourceObject` - the source object: `sourceProperty` - the source property: `targetObject` - the target object: `targetProperty` - the target property **Returns:**: an `AutoBinding` that binds the source object to the target property of the target object **Throws:**: `java.lang.IllegalArgumentException` - if the update strategy, source property or target property is `null`

---


### createAutoBinding

```
public static <SS,SV,TS,TV> AutoBinding<SS,SV,TS,TV> createAutoBinding(AutoBinding.UpdateStrategy strategy,
                                                                       SS sourceObject,
                                                                       Property<SS,SV> sourceProperty,
                                                                       TS targetObject,
                                                                       Property<TS,TV> targetProperty,
                                                                       java.lang.String name)
```

:   Creates a named instance of `AutoBinding` that binds a property of a source object to a property of a target object.

    :   **Parameters:**: `strategy` - the update strategy for the binding: `sourceObject` - the source object: `sourceProperty` - the source property: `targetObject` - the target object: `targetProperty` - the target property: `name` - a name for the binding **Returns:**: an `AutoBinding` that binds the source object to the target property of the target object **Throws:**: `java.lang.IllegalArgumentException` - if the update strategy, source property or target property is `null`


---


|  |  |  |
| --- | --- | --- |
| **Overview** \| **Package** \| **Class** \| **Use** \| **Tree** \| **Deprecated** \| **Index** \| **Help** | |  |
| **PREV CLASS**   **NEXT CLASS** | **FRAMES**    **NO FRAMES**     **All Classes** |  |
| SUMMARY: NESTED \| FIELD \| CONSTR \| METHOD | DETAIL: FIELD \| CONSTR \| METHOD |  |


---
